# Supplementary material for: Defining destigmatizing design guidelines for use in sexual health-related digital technologies: A Delphi study
Source: PLOS Digit Health. 2023 Jul 12;2(7):e0000223. doi: 10.1371/journal.pdig.0000223 (PMC10337937; doi:10.1371/journal.pdig.0000223)
Supplement: S1 Table — (DOCX) [file pdig.0000223.s001.docx]

**S1 Table. Design guideline revisions at Rounds 1 and 2**

| **Design Guidelines revised after Only 1 Round** | | | | | | | |
| --- | --- | --- | --- | --- | --- | --- | --- |
| **ID** | **Original Guideline** | | | **ID** | **Revised Guideline after Round 1** | | |
| DG9 | Avoid using language that seeks to blame, or cast moral judgment on people living with or infected by a condition | | | DG9.1 | Avoid using language that seeks to blame or judge people living with a condition | | |
| DG10 | Provide opportunities for selective and voluntary disclosure of status to provide emotional connection and support | | | DG10.1 | For websites that provide interpersonal connection, provide opportunities for selective and voluntary disclosure of status to provide emotional connection and support | | |
| DG11 | Provide interpersonal contacts for people to seek direct online counseling and other psychological support | | | DG11.1 | Provide contact information for people to seek online counselling or other psychological support | | |
| DG16 | Include information that seeks to correct wrong perceptions and myths surrounding a particular condition to enable users to reject negative and inaccurate beliefs | | | DG16.1 | Include information that corrects myths about a condition, to enable users to get accurate information | | |
| DG17 | Educate users about stigma by providing factual and plain language information that normalizes and de-stigmatises sexual health-related conditions | | | DG17.1 | Provide factual and plain language information that normalizes and de-stigmatises sexual health-related conditions | | |
| DG18 | Use positive, credible, and diverse language and images | | | DG18.1 | Selection of language/images should be done in consultation with the community to ensure diversity | | |
| DG19 | Use images and pictures of people from a variety of backgrounds who have experienced the condition | | | DG19.1 | Use images of people from diverse ethnicity, age, and gender identity who have experienced the condition | | |
| DG21 | Use personal stories of renowned and relatable personalities who have experienced the condition | | | DG21.1 | Use personal stories of people who have experienced the condition. e.g. celebrities, or lesser-known personalities | | |
| DG22 | Provide information on the fundamental rights of people affected by or living with the condition. e.g., Rights of persons living with HIV/AIDS | | | DG22.1 | Consider including links to information on the fundamental rights of people affected by or living with the condition | | |
| DG23 | Use inclusive language such as “we, our, or us” and “you are not alone” | | | DG23.1 | Use inclusive language that is sensitive to contextual issues. e.g. partner instead of husband/wife, person instead of woman/man | | |
| DG26 | Include a video or written testimonial of real people to talk about stigma and their experiences with the condition | | | DG26.1 | Include videos/testimonials that center on people’s experiences with the condition, including stigma | | |
| **Design Guidelines revised after two Rounds** | | | | | | | |
|  | **Original Guideline** | **ID** | **Revised Guidelines after R1** | | | **ID** | **Revised Guidelines after R2** |
| DG5 | Logos, icons, and terminologies should be made subtle so that they do not immediately draw the users’ or bystanders’ attention to sexuality | DG5.1 | Use logos, icons, and terminologies that clearly depict the topic for the website | | | DG5.2 | Use logos, icons, and terminologies that help to explain the topic for the website as long as privacy issues are considered (e.g., shoulder surfing) |
| DG7 | Avoid conveying negative experiences associated with a condition and rather emphasize positive messaging | DG7.1 | Ensure a balanced presentation of information by presenting both positive and negative experiences about a sexual health issue | | | DG7.2 | Ensure a balanced presentation of information by highlighting more on positive experiences but not lying by omission |
| DG8 | Use humour in a very positive and sensitive manner to lessen the fear and seriousness associated with a condition | DG8.1 | Use humour only if appropriate and do extensive user testing | | | DG8.2 | Use humour only if appropriate and do extensive user testing to confirm context-specific appropriateness |
| DG12 | Include links to other credible sites and contact information for users to seek further information | DG12.1 | Use hyperlinks to direct users to other websites that provide more information | | | DG12.2 | Consider using hyperlinks to other websites that provide more information |
| DG13 | Ensure appropriate flexibility of web features to allow a user to hide certain content/images that they find offensive | DG13.1 | Use images that foster kindness, happiness, connection, support, engagement, respect, and community | | | DG13.2 | Use pictures that depict kindness, connection, support, engagement, respect, and community, as long as it does not promote perceptions of being emotionally manipulated |
| DG15 | Expose people to a range of messaging that addresses different aspects of stigma | DG15.1 | Include a range of information that touches on different aspects of the condition | | | DG15.2 | Include a range of evidence-based information that touches on different aspects of the condition |
| DG25 | Provide mechanisms for creating online advocacy groups | DG25.1 | Provide a secure link or a section for creating online support groups | | | DG25.2 | Provide a secure link for provider-moderated online support groups |

Note: Items DG3, DG4, DG6, DG14, DG20, and DG24 were dropped in Round 1 due to overlap
