# Supplementary material for: Defining destigmatizing design guidelines for use in sexual health-related digital technologies: A Delphi study
Source: PLOS Digit Health. 2023 Jul 12;2(7):e0000223. doi: 10.1371/journal.pdig.0000223 (PMC10337937; doi:10.1371/journal.pdig.0000223)
Supplement: Attachment — Submitted filename: Comments on Abdulai et al 2023.pdf [file pdig.0000223.s002.pdf]

Dear authors,

Thank you for submitting the study: „Defining Destigmatizing Design Guidelines for Use in Sexual Health-Related Digital Technologies: A Delphi Study“.

The study describes the development of 19 guidelines for the destigmatizing design of digital sexual health solutions using the Delphi method. In three rounds, 13 researchers were consulted. The topic has great practical relevance for development teams and the method is quite suitable for developing those guidelines. The manuscript is written in a concise and understandable manner. Enclosed are minor issues that should be addressed prior to publication.

Kind regards

#	Manuscript passage	Comment
1	**Introduction** [Whereas public stigma manifests…]	The difference between internalized and public stigma is not quite clear. I would recommend to rephrase this sentence and thereby consider:<br>• Who is the source of public stigmatisation?<br>• At someone → at a group (with negative attributes)
2	**Introduction** [Despite global efforts…to hamper progress]	Please add a literature reference.
3	**Paticipant recruitment**	You have only recruited researchers as experts and no experts from the field (e.g. a social worker with 10 years of experience in HIV/AIDS counselling could also provide information on appropriate guidelines). Perhaps you can add this to your limitations or the conclusion passage.
4	**Data Collection** […on a scale of 1-7, where 7 indicate the strongest level of agreement.]	Please add more information about the scale range. (likert-scaled; 1= strong disagreemant, 3=neutral, 7=strong agreement)
5	**Data Collection** [Additional guidelines generated from participants' comments and suggestions...]	Please add more information about the process of generating new guidelines (who, how many researchers)
6	**Data Analysis** […the panel should agree on its clarity and usefulness...]	Please add: Agreement means values ≥4(?)
7	**Data Analysis** [The final absolute score could range from 0-1 where a higher score (typically above 0.2, or 20%) indicates wide variations in response options between the two rounds and a lower score...]	Please add: lower score (typically lower than 0.8(?), or 80%(?))
8	**Data Analysis, Delphi Results**	Make sure that you define what "overlapping" guidelines are
9	**Delphi Results** and **Figure 1**	Please check the numbers and make sure that each value from figure 1 can be found in the text and vice versa. Currently it is confusing. For instance "a total of 18 new guidelines were obtained…" can't be identified in figure 1 clearly

		or the 5 discarded guidelines from R1 from figure 1 are not mentioned in the text.
**10**	**Figure 1**	Please make more clear which guidlines retain, which are newly suggested etc. Currently it is quite confusing that for instance in R1 *12+16+24+5+4>26*.<br>Better would be (R1): 28 guidelines<br>a) 12 achieved consenus<br>→ 4 retained for final guidelines<br>→ 2 (or 3?) revised in R2<br>→ 5 discarded<br>b) 16 no consensus<br>→ 16 revised in R2<br>c) 8 Newly sugested<br>→ R2
**11**	**Table 1** [DG3]	Revised → Dropped (?) in R1 due to overlap
**12**	**Table 3**	Remove line between DG26.1 and DG27
**13**	**Discussion** [These principles include DP16 (i.e., Include information that corrects myths about the condition), and NDP35 (i.e., Provide factual and plain language information).]	Please explain or do not abbreviate *DP* and *NDP*
**14**	**Limitations** [Further studies should convene adopt a focus group among experts as well as patients to further understand how the design guidelines can be applied.]	A better term for “patients” would be “users” or “people who receive the digital health solution”.
**15**	**Conclusion** [The guidelines developed in this study are often not considered by development teams…]	Since the guidelines have only just been developed, they can in principle not yet be applied. But the principles may already be (partly) taken into account by developers. Therefore: Can you really make this assumption? Then it should be made clear that this statement emerges, for example, from the expert opinions.
**16**	**Conclusion**	Dissemination plans for the guidelines could be given and details on how they will be made available to development teams could be provided.
**17**	**Supplemental Table S1.**	Move DG26 after DG23 or explain why it is mentioned earlier
