# Supplementary material for: Defining destigmatizing design guidelines for use in sexual health-related digital technologies: A Delphi study
Source: PLOS Digit Health. 2023 Jul 12;2(7):e0000223. doi: 10.1371/journal.pdig.0000223 (PMC10337937; doi:10.1371/journal.pdig.0000223)
Supplement: Attachment — Submitted filename: RESPONSE TO REVIEWS COMMENTS.docx [file pdig.0000223.s003.docx]

Dear editor and reviewers,

We are pleased to resubmit the revised version of our manuscript title ***Defining Destigmatizing Design Guidelines for Use in Sexual Health-Related Digital Technologies: A Delphi Study****.*  We highly appreciate the valuable comments of the reviewers. We believe that the manuscript is substantially improved after making the suggested edits. Following this note are the reviewers’ comments with our responses, including how and where the text was modified. The revision has been developed in consultation with the coauthors, and each author has approved the final form of this revision.

Thanks, and Best Regards!

Yours Sincerely

| Comments | Response |
| --- | --- |
| Reviewer 1 |  |
| Please confirm whether "ethics committee" should be capitalized as part of the name of the UBC Research Ethics Board. | We have capitalized "Ethics Committee" |
| On page 11, I think the word "indicate" should be plural when you describe the scale, i.e. "...where 7 indicates..." | Based on the comments of reviewer 2, we have replaced this statement with a complete description of the 7-point Likert scale and this revision addresses this comment |
| Pages 20-21, in Table 3, consider shifting the first column so the framework category titles are not hyphenated. | We have made these changes and the trauma-informed care framework categories are no longer hyphenated. |
| On page 22, please format the references for Friedman et al., Hood & Friedman, and Rocha-Jimenez are formatted consistent with the other references in the manuscript. | We have formatted these references to be consistent with the reference format of the manuscript |
| On page 23, clarify whether "NDP35" is correct | Many thanks for spotting this error. The correct design guideline is DG17.1 (i.e., Provide factual and plain language information that normalizes and de-stigmatizes sexual health-related conditions). We have made these changes accordingly. |
| On page 23, confirm whether you need the word "based" at the end of the sentence ending with "...the predominant use of educational messages in stigma reduction campaigns based [30]." | We have removed this word and the sentence now reads well |
| On page 24 in the limitations section, I think you can remove the word "adopt" before the phrase "... a focus group among experts... | We have removed this word accordingly |
| Reviewer 2 |  |
| **Introduction:** The difference between internalized and public stigma is not quite clear. I would recommend to rephrase this sentence and thereby consider:   - Who is the source of public stigmatisation? - At someone →at a group (with negative attributes) | To make the differences between internalized and public stigma quite explicit, we have rephrased the statement to read as  “*Internalized stigma occurs when a person perceives their situation as a negative attribute with resulting decreases in self-esteem and self-worth While public stigma manifests as overt discriminatory practices targeted at a group with negative attributes*” |
| **Introduction:** Add a reference to (Despite the global efforts…. to hamper progress) | We have provided a reference to this statement |
| **Participant Recruitment:** You have only recruited researchers as experts and no experts from the field (e.g. a social worker with 10 years of experience in HIV/AIDS counselling could also provide information on appropriate guidelines). Perhaps you can add this to your limitations or the conclusion passage. | We have added this concern as a limitation by stating that  “*Instead of recruiting only people with research expertise on the topic, we also believed that the design guidelines could have benefited from a review by experts in the field of sexual health practice*” |
| **Data Collection:** Please add more information about the scale range. (likert-scaled; 1= strong disagreement, 3=neutral, 7=strong agreement) | We have provided a complete description of the 7-point Likert scale by stating that  “*The participants rated the clarity and usefulness of each guideline in addressing sexual health-related stigma via digital platforms on a scale of 1-7, where 1 = strongly disagree, 2 = disagree, 3 = mildly disagree, 4 = neutral, 5 = mildly agree, 6 = agree, and 7 = strongly agree*” |
| **Data Collection:** Please add more information about the process of generating new guidelines (who, how many researchers) | We have added more information on how new items were generated from Round 1 and Round 2 by stating under the data collection that….  “*Additional design guidelines were also generated from participants comments and suggestions from Round 1” …. “Additional guidelines were also generated from* *Round 2* *and the new items as well as items not meeting consensus were presented in Round 3”* |
| **Data collection:** Please add: Agreement means values ≥4(?) | We have added the agreement value in the as the last sentence of the section under data collection by stating that…  “*An item was considered as having been agreed upon if it obtained a mean value ≥ 5 on the Likert scale (i.e., overall value greater than “mildly agree”)”* |
| **Data Analysis:** Please add: lower score (typically lower than 0.8(?), or 80%(?)) | We have added the value for the lower scores by stating…..  *“(i.e., scores typically lower than 20%)”* |
| **Data Analysis:** Make sure that you define what “overlapping” guidelines are | Instead of saying that items overlapped, we have clarified that by saying the content of such items were found in other guidelines. We stated this by indicating in the first paragraph of the section under the Delphi Results that…  “*5 were dropped* *(i.e., DG3, DG4, DG6, DG14, DG20, and DG24) because the content was found in other guidelines.”* |
| **Delphi Results:** Please check the numbers and make sure that each value from figure 1 can be found in the text and vice versa. Currently it is confusing. For instance, “a total of 18 new guidelines were obtained” can’t be identified in the figure. or the 5 discarded guidelines from R1 from figure 1 are not mentioned in the text. | We have revised the figure and the description of the figure to be consistent with each other. Now the description in the text can be seen and verified from the figure. |
| **Table 1 [DG3]** Revised → Dropped (?) in R1 due to overlap | We have changed “revised” to “dropped” for DG3 in Table 1 |
| **Table 3 Remove** line between DG26.1 and DG27 | We have removed the line accordingly |
| **Discussion:** Please explain or do not abbreviate *DP* and *NDP* | DP and NDP were not the correct IDs for the design guidelines. We have replaced them with the correct IDs. That is, DP16 is replaced with DG16.1 and NDP35 is replaced with DG17.1 |
| **Limitations:** A better term for “patients” would be “users” or “people who receive the digital health solution” | These changes have been made accordingly |
| **Conclusion:** Since the guidelines have only just been developed, they can in principle not yet be applied. But the principles may already be (partly) taken into account by developers. Therefore: Can you really make this assumption? Then it should be made clear that this statement emerges, for example, from the expert opinions. | We have revised this statement by replacing it with what we originally thought to be the intended meaning by stating that…..  “*Design guidelines that transcends beyond the usual functionalist considerations (such as those similar to ours) are often not considered by development teams, yet* *considered critical for ensuring emotional comfort*” |
| **Conclusion:** Dissemination plans for the guidelines could be given and details on how they will be made available to development teams could be provided. | We have provided a dissemination and uptake strategy by stating that  “*For the design guidelines to be successful in addressing stigma, project managers or organizations that wish to embark of digital health projects for sensitive health topics like sexual health should consider making this reference guide an essential part of the project contract when engaging with the development team. This way, digital health developers can ensure that the final product will strive to prevent stigma*” |
| **Supplemental Table 1:** Move DG26 after DG23 or explain why it is mentioned earlier | We have moved DG26 after DG23 |
